# The hidden function of egg white antimicrobials: egg weight-dependent effects of avidin on avian embryo survival and hatchling phenotype

**DOI:** 10.1242/bio.031518

**Published:** 2018-03-14

**Authors:** Eva Krkavcová, Jakub Kreisinger, Ludmila Hyánková, Pavel Hyršl, Veronika Javůrková

**Affiliations:** 1 Department of Zoology, Charles University, Viničná 7, 128 44, Prague, Czech Republic; 2 The Czech Academy of Sciences, Institute of Vertebrate Biology, Květná 8, 603 65 Brno, Czech Republic; 3 Department of Genetics and Breeding of Farm Animals, VÚŽv.v.i., Přátelství 815, 104 00, Prague-Uhříněves, Czech Republic; 4 Department of Experimental Biology, Faculty of Science, Masaryk University, Kotlářská 2, 611 37, Brno, Czech Republic; 5 Department of Animal Husbandry, Czech University of Life Sciences, Kamýcká 129, 165 00 Prague – Suchdol, Czech Republic

**Keywords:** Albumen, Maternal effects, Antimicrobials, Avidin-biotin complex, Embryogenesis, Plasma complement

## Abstract

Avidin is a key egg white antimicrobial protein with strong binding capacity for biotin, an essential growth and immune cell precursor. As such, it is assumed to have a pronounced, though still poorly explored, effect on hatchling phenotype. We tested the effect of experimentally increased egg white avidin concentration (AVIDIN+) on hatching success, chick morphology, post-hatching growth performance and innate immune function in a model bird, Japanese quail (*Coturnix japonica*). Probability of embryo survival in the late embryonic phase increased with increasing egg weight in control eggs, but not in AVIDIN+ eggs. Chicks hatching from lighter AVIDIN+ eggs had a shorter tarsus than chicks hatching from heavier AVIDIN+ eggs. This suggests that an increase in egg white avidin favours embryo survival in lighter eggs during late embryogenesis, but at the expense of reduced structural body size. Plasma complement activity in 6-day-old AVIDIN+ chicks decreased with increasing body mass and tarsus length; the opposite was observed in control chicks, implying that the later post-hatching innate immune function of larger chicks was compromised by an increase in egg white avidin concentration. Here, we document an important role of egg white antimicrobials in maintenance of embryo viability, avian hatchling morphology and immune phenotype.

## Abstract (precis)

**Summary:** This is the first experimental study to evaluate the effects of increased concentration of egg white antimicrobial protein avidin on embryo viability, hatchling morphology and immune phenotype in birds.

## INTRODUCTION

In birds, parental investments linked with maternal deposition of different substances into the egg during oogenesis are assumed to directly affect brood and offspring quality (Mousseau and Fox, 1998; Gil, 2003; Navara and Mendonca, 2008; Hasselquist and Nilsson, 2009). Most studies to date have focused on maternal compounds that are deposited into the yolk during oogenesis (e.g. immunoglobulins, steroids, carotenoids) and have a direct effect on offspring prosperity during embryonic and post-embryonic development (Schwabl, 1993, 1996; Groothuis et al., 2005). In comparison, little attention has been paid to the effects of maternally deposited egg white biomolecules on hatchling development and fitness-associated traits.

Egg white acts as both a thermal and mechanical defence mechanism for the embryo, is an important source of water and nutrition during development, and contains a broad spectrum of proteins that play an essential role during embryo development (Burley and Vadehra, 1989). Experimental studies have shown that the amount of egg white may directly affect offspring phenotype by decreasing hatchling body weight or causing developmental asymmetries (Finkler et al., 1998; Bonisoli-Alquati et al., 2007). Egg white proteins are also thought to provide an effective defence against microbial trans-shell infection. Both the alkaline pH (Grizard et al., 2015) and the presence of antimicrobial proteins (Shawkey et al., 2008; Horrocks et al., 2014) have been shown to significantly reduce the growth of microorganisms (Board and Fuller, 1974) able to penetrate the eggshell and fundamentally affect egg hatchability (Pinowski et al., 1994) and/or offspring phenotype (Javůrková et al., 2014). In addition, many egg white proteins are thought to be important modulators of embryogenesis and immune phenotype; though few correlative studies (Saino et al., 2002, 2007; Bonisoli-Alquati et al., 2010; D′Alba et al., 2010) and only one experimental study (Javůrková et al., 2015) have examined the effect of egg white proteins on the development and post-hatching phenotype of birds. Many egg white proteins also have binding sites specific to metal ions, such as Fe^3+^, or to vitamins, such as biotin, riboflavin or thiamine (Muniyappa and Adiga, 1979; White and Merrill, 1988). Although the binding of these essential molecules to specific proteins prevents their use in the growth and proliferation of many pathogenic microorganisms, their presence is probably more important for proper development of the embryo (Bonisoli-Alquati et al., 2010; Giansanti et al., 2012; Javůrková et al., 2015).

Avidin is a tetrameric protein that occurs in the eggs of all oviparous vertebrates. Each avidin monomer can reversibly bind water-soluble biotin (also called vitamin H) with high affinity and specificity (Board and Fuller, 1974). The binding of biotin to egg white avidin results in its unavailability for microorganisms and thereby prevents their proliferation. As such, avidin can be considered bacteriostatic (Wellman-Labadie et al., 2008). Biotin is involved in many metabolic processes, including production of fatty acids or fats and amino acid metabolism, and has a significant effect on gene expression and cell proliferation and differentiation during embryogenesis (Dakshinamurti, 2005; Zempleni et al., 2009). Accordingly, an increase in the concentration of egg white avidin may favour a developing embryo by increasing its resistance to pathogens; however, avidin's ability to bind the biotin growth precursor may have a negative impact on offspring development and fitness.

In this study, we examine the counteractive action of egg white avidin by studying the effect of avidin manipulation on egg hatchability and morphological traits, and growth and innate immune function of hatchlings, using Japanese quail (*Coturnix japonica*) as a precocial model species of bird. We test two main hypotheses: (i) that eggs with a higher concentration of egg white avidin show improved hatchability and embryo viability, and (ii) that chicks hatched from eggs with elevated concentration of egg white avidin display reduced growth, morphometric characteristics and innate immune function compared to chicks from control eggs. There is strong interspecific (Cucco et al., 2009; Bonisoli-Alquati et al., 2010) as well as intraspecific (D′Alba et al., 2010) variability in the concentration of egg white avidin in birds. To assess sensitivity of embryos to experimentally increased concentration of avidin in eggs that vary in initial concentration of this protein, we used three separate lines of Japanese quail with different concentrations of avidin in egg whites. Thus, we were able to robustly test potential consequences of variable intraspecific maternal allocation of avidin into the eggs on embryo survival and offspring phenotype.

## RESULTS

### Hatching success and post-hatching survival

We found no effect of egg white avidin manipulation on overall hatching success or embryo mortality in either early or late developmental stages (Table 1; mean±s.e. probability of hatching=0.65±0.04 and 0.58±0.04, and mean±s.e. probability of surviving early incubation=0.90±0.02 and 0.88±0.03 for AVIDIN+ versus control PBS+ eggs, respectively). Overall mean (±s.e.) hatching success for the HG, K and LG strains was 0.53±0.05, 0.61±0.05 and 0.69±0.04, respectively. A similar pattern was observed when analysing late embryonic mortality: 0.83±0.04, 0.91±0.03 and 0.92±0.02 for the HG, K and LG lines, respectively.

**Table 1.**
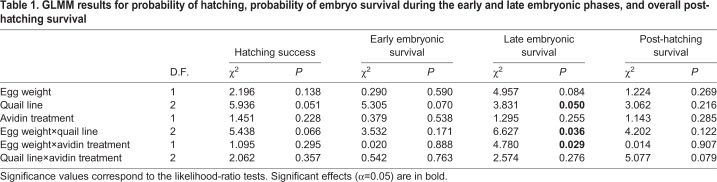
**GLMM results for probability of hatching, probability of embryo survival during the early and late embryonic phases, and overall post-hatching survival**

**Fig. 1.**
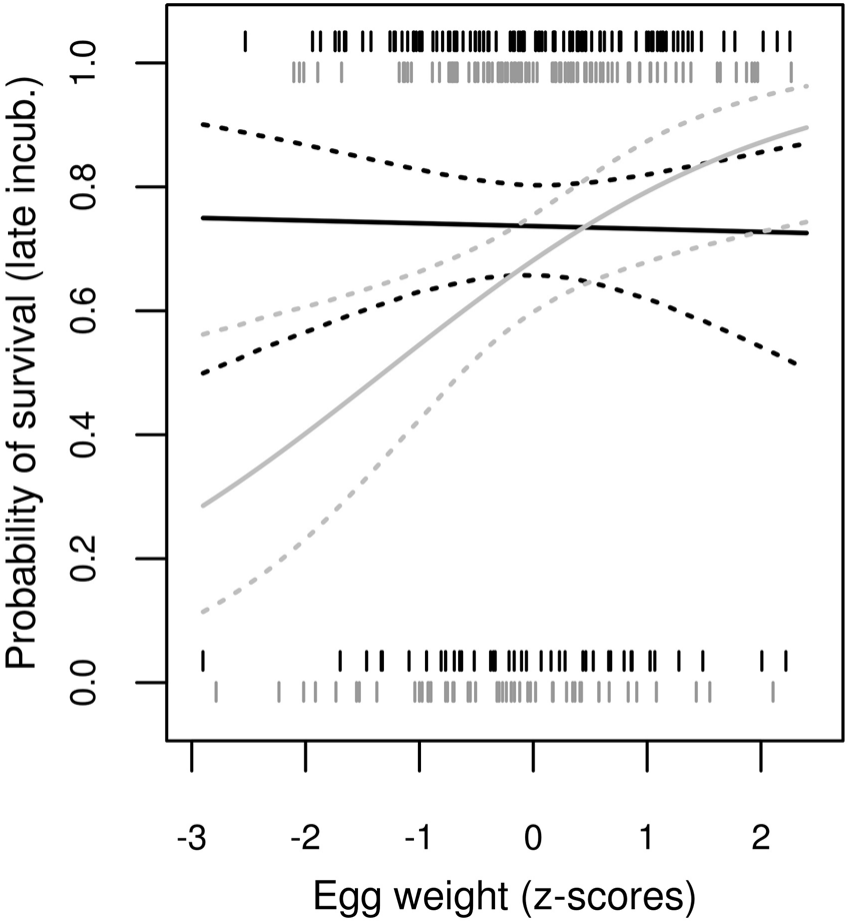
**Interactive effect of egg white avidin treatment and egg weight on the probability of embryo survival during the late embryonic phase.** The PBS+ (control) and AVIDIN+ groups of eggs are plotted in grey and black, respectively. Model predictions and 95% bootstrap confidence intervals based on the GLMM were computed using the ez package in R.

We did find a significant interactive effect of avidin treatment versus egg weight on the probability of survival during the late embryonic phase (Table 1). Although late embryonic phase survival for AVIDIN+ eggs was unaffected by egg weight [Separate generalized linear mixed model (GLMM) for AVIDIN+ eggs: Logit slope±s.e.=0.038±0.178, delta degrees of freedom (Δ D.F.)=1, χ2=0.046, *P*=0.830], we observed a marked positive association between egg weight and late embryonic survival in the control PBS+ eggs (Separate GLMM for PBS+ eggs: Logit slope±s.e.=0.466±0.197, Δ D.F.=1, χ^2^=5.963, *P*=0.015; Fig. 1).

Post-hatching survival rates were relatively high during the experiment, with only 14.7% of chicks failing to survive the experimental period. Mortality was not associated with line identity, egg weight, avidin treatment or any two-way interaction of these variables (Table 1).

### Early post-hatching morphological traits

**Fig. 2.**
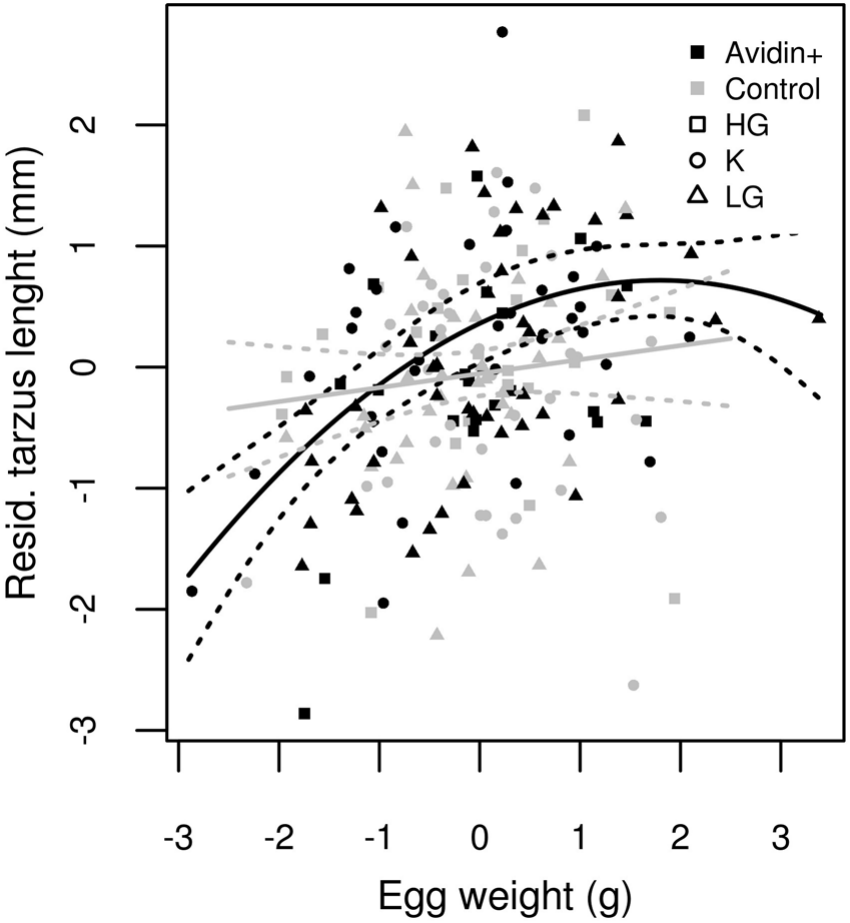
**Interactive effect of egg white avidin treatment and egg weight on the relationship between residual tarsus length and egg weight in freshly hatched quail chicks.** The PBS+ (control) and AVIDIN+ groups are plotted in grey and black, respectively. Predictions for AVIDIN+ eggs were based on a quadratic GLMM and those for control PBS+ eggs using a linear GLMM. Model predictions and 95% bootstrap confidence intervals were computed using the ez package in R.

Avidin treatment had no significant effect on body weight or residual tarsus length in freshly hatched chicks (Table 2). The only variable that showed a significant positive effect on both body weight and residual tarsus length in freshly hatched chicks was egg weight (Table 2). Furthermore, GLMM uncovered a marginally non-significant effect in the interaction between egg weight and avidin treatment on residual tarsus length (Table 2). Visual inspection of the corresponding plots suggests that there is no apparent relationship between egg weight and residual tarsus length for PBS+ chicks. In contrast, AVIDIN+ chicks originating from lighter eggs tend to have a shorter tarsus length than expected based on body weight; the difference was not seen in hatchlings from larger eggs (Fig. 2). Replacing the linear relationship between residual tarsus length and egg weight with a quadratic term significantly decreased the residual deviance explained (Δ D.F.=1, χ^2^=6.738, *P*=0.01), while the egg weight versus avidin treatment interaction remained marginally non-significant (Δ D.F.=1, χ2=3.704, *P*=0.054). A quadratic model fitted separately for AVIDIN+ chicks was more informative than the simple linear model (Δ D.F.=1, χ^2^=6.915, *P*=0.009) and provided highly significant results compared to the null model (Δ D.F.=1, χ^2^=25.816, *P*<0.0001). On the other hand, the null model failed to provide a better fit than the linear relationship between PBS+ chicks (Δ D.F.=1, χ^2^=1.172, *P*=0.279). Moreover, inclusion of a quadratic term into the linear model for PBS+ chicks did not decrease residual deviance significantly (Δ D.F.=1, χ^2^=1.178, *P*=0.278).

**Table 2.**
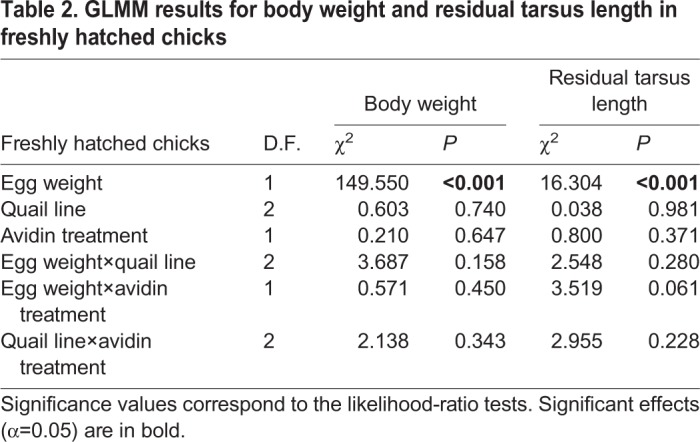
**GLMM results for body weight and residual tarsus length in freshly hatched chicks**

### Late post-hatching morphological traits

To assess variation in morphometric parameters over the 15-day experiment, we applied strain-specific z-score scaling for each of the six age classes (see Materials and Methods for details). Thus, the transformed morphometric data had a zero mean and the same variance throughout the experiment. This step considerably reduced analysis complexity, as the data no longer require explicit modelling of growth curves using nonlinear models. We found no support for the effect of avidin treatment or its interactions with other variables when modelling variation in z-score transformed body weight and residual tarsus length (Table 3). The only variable that showed a significant level of support in these models was the interaction of lipopolysaccharide (LPS) treatment with chick age.

**Table 3.**
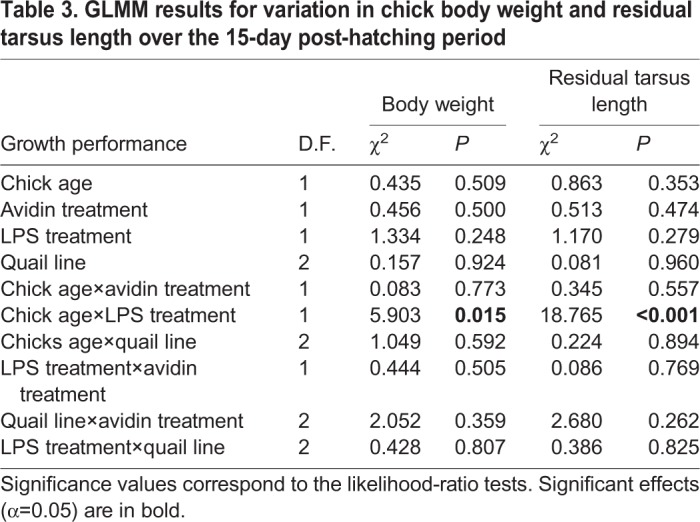
**GLMM results for variation in chick body weight and residual tarsus length over the 15-day post-hatching period**

### Effect of LPS treatment and total plasma complement activity

GLMM results indicate LPS treatment as significantly compromising increase in body weight and residual tarsus length in chicks between 3 and 6 days after hatching (Table 4). The mean (±s.e.) increase in tarsus length in PBS-treated control chicks was 4.36±0.11 (pre- and post-treatment averages were 20.88±0.18 mm and 25.24±0.26 mm, respectively), but just 3.68±0.11 in LPS-treated chicks (pre- and post-treatment averages were 21.09±0.20 mm and 24.77±0.28 mm, respectively). Similarly, body weight between 3-day-old and 6-day-old chicks increased by 18.49±0.66 g in PBS-treated control chicks (pre- and post-treatment averages were 22.85±0.77 g and 41.34±1.34 g, respectively), but only by 15.39±0.60 g in LPS-treated chicks (pre- and post-treatment averages were 23.36±0.83 and 38.75±1.36, respectively). However, there was no difference in LPS treatment response in freshly hatched AVIDIN+ and PBS+ chicks, the interaction between avidin and LPS treatment being non-significant (Table 4). The effect of all other variables included in the model was also non-significant.

**Table 4.**
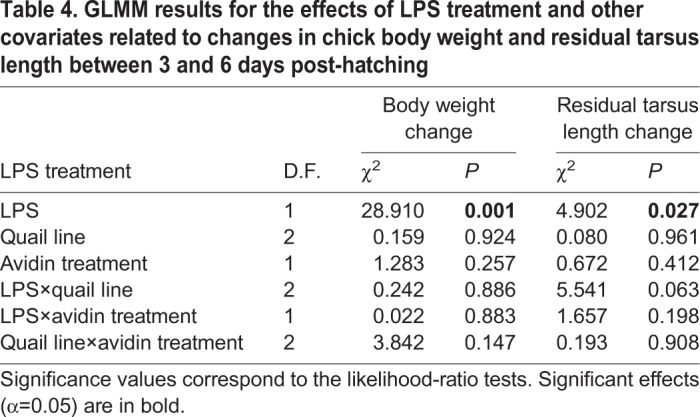
**GLMM results for the effects of LPS treatment and other covariates related to changes in chick body weight and residual tarsus length between 3 and 6 days post-hatching**

**Fig. 3.**
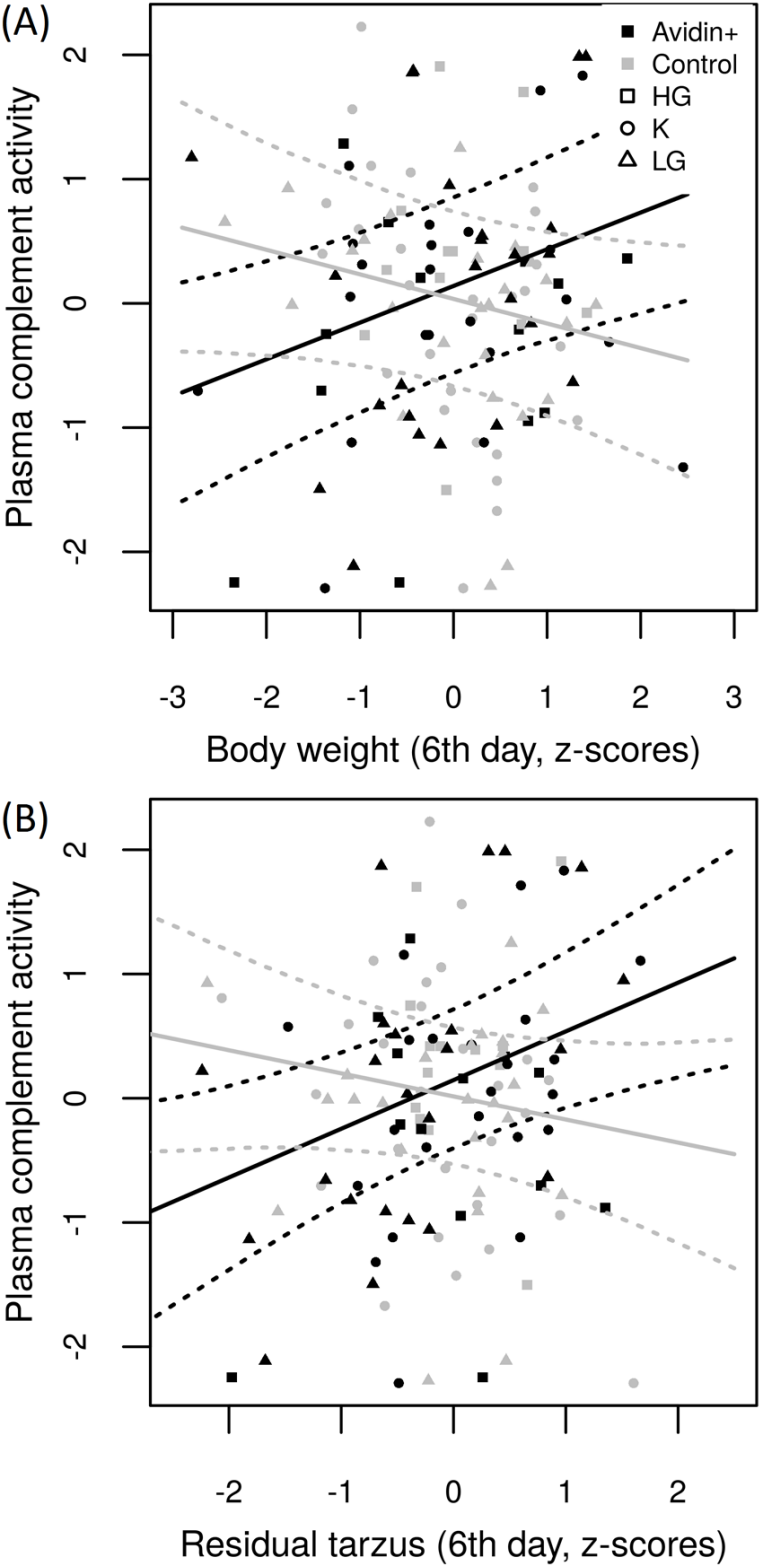
**Relationship between total plasma complement activity and (A) body weight and (B) residual tarsus length for 6-day-old quail chicks.** The PBS+ and AVIDIN+ groups are plotted in grey and black, respectively. Please note that there is an inverse scaling of plasma complement activity (i.e. a shorter time represents higher total plasma complement activity). Model predictions and 95% bootstrap confidence intervals based on GLMM were computed using the ez package in R.

Because data on complement activity were obtained for 125 chicks (due to plasma sample loss), and LPS treatment had no effect on total plasma complement activity (Univariate GLM: Δ D.F.=1, χ^2^=0.096, *P*=0.757), the main and interactive effects of LPS treatment were excluded from the GLMM to maintain a reasonable ratio between sample size and number of explanatory variables. We found highly significant support for the interactive effect of avidin treatment and all morphometric parameters on plasma complement activity (Table 5). There was a positive association between inversely scaled plasma complement activity and body weight (slope±s.e.=0.301±0.101, Δ D.F.=1, χ2=8.087, *P*=0.004), and residual tarsus length (slope±s.e.=0.270±0.105, Δ D.F.=1, χ2=6.140, *P*=0.013) in AVIDIN+ chicks, suggesting that total plasma complement activity was compromised in larger chicks after avidin manipulation (Fig. 3A). On the other hand, we observed a negative marginally non-significant association between total plasma complement activity and body weight (slope±s.e.=−0.233±0.118, Δ D.F.=1, χ2=3.735, *P*=0.053), and a non-significant relationship between plasma complement activity and residual tarsus length, in PBS+ chicks (slope±s.e.=−0.117±0.115, Δ D.F.=1, χ2=1.023, *P*=0.312; Fig. 3B). Nevertheless, neither the main effect of avidin treatment nor other explanatory variables were associated with total plasma complement activity.

**Table 5.**
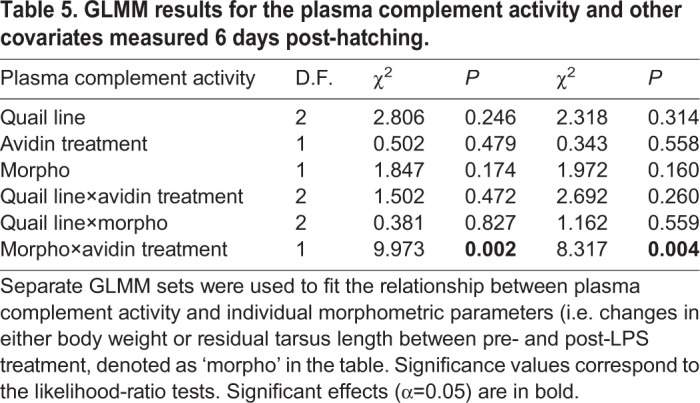
**GLMM results for the plasma complement activity and other covariates measured 6 days post-hatching.**

## DISCUSSION

In this study, we provide the first experimentally documented evidence for the critical function of the antimicrobial protein avidin in avian embryogenesis. Specifically, we noted an increased probability of embryo survival during the late embryonic developmental phase, with increasing egg weight in eggs with normal concentrations of egg white avidin. In contrast, the probability of late embryonic phase survival for avidin-treated eggs remained constant, regardless of egg weight. In other words, the probability of survival during the late embryonic phase was greater for embryos developing in lighter eggs following supplementation of egg white with avidin compared to embryos developing in lighter eggs without supplementation. The question arises: How does supplementation with avidin in eggs with lower egg mass improve embryo survival in the late embryonic phase?

The positive effect of egg mass on embryo survival has been well documented in both commercial (Ishaq et al., 2014) and wild breeding birds (D'Alba and Torres, 2007; Krist, 2011). This is to be expected, as proper embryo development is highly dependent on the amount of egg nutrient proportionally distributed in the yolk and egg white (Palmer and Guillette, 1991; Groothuis and Engelhardt, 2005). Because absolute mass of all egg components (i.e. yolk, egg white) is higher in larger eggs (Williams, 1994; Hill, 1995), embryos in larger/heavier eggs have the advantage of a higher proportion of nutrients. It is worth noting, however, that the content of yolk and egg white in different sized eggs is not proportionally constant in birds, and that positive or negative allometry exists in yolk/egg white content across taxa. A relatively higher proportion of egg white has been documented in larger/heavier eggs for most altricial birds, whereas precocial birds tend to have relatively more yolk content in larger eggs (Williams, 1994; Hill, 1995). Consequently, the improving effect of egg weight on embryo survival during the late embryonic phase documented in our study is most probably related to the increased nutrient content in the larger egg yolks of heavier quail eggs. This assumption is supported by the importance of the amount of macromolecules in egg yolk during late stages of embryo development (Moran, 2007), while earlier in embryonic development, the properties of the egg white are more important for embryonic survival (Christensen, 2001).

There are many possible scenarios explaining the positive effect of avidin treatment on embryo survival in lighter eggs during the late phase of embryogenesis. The most plausible explanations are related to mechanisms linked with the binding capacity of avidin to other biomolecules. In addition to previously documented strong binding affinity of avidin for biotin (Green, 1975), each avidin monomer has one N-linked carbohydrate side chain, and 10% of avidin mass could be explained by the carbohydrates (mannose and N-acetylglucosamine) content (DeLange, 1970; Bruch and White, 1982). Although bird eggs are rich in carbohydrates (Burley and Vadehra, 1989), carbohydrates play an essential role in embryo and hatchling nutrition (Moran, 2007), and are developmentally important during the both early and late embryonic phases, especially in precocial birds (Foye et al., 2007). Sunny and Bequette (2010) noted that gluconeogenesis rate (i.e. the forming of glucose *de novo*, especially from amino acids) is greater in chick embryos developing from smaller eggs. They also observed greatly reduced concentrations of gluconeogenic amino acids in the blood of 12- to 18-day-old embryos from small eggs, probably related to reduced embryonic growth resulting from utilisation of these amino acids during increased gluconeogenesis. We therefore suggest that enrichment of egg white with glycoprotein in the form of avidin (i.e. a change in the proportion of carbohydrates to amino acids) may improve carbohydrate sources for embryo during the late embryogenesis and, subsequently, increases the probability of embryo survival during this phase. However, although there has yet to be any experimental testing of such mechanisms in avian embryogenesis, further studies are needed, to test whether and how enrichment of egg white with avidin increases survival of embryo during different phases of embryo development in precocial and/or altricial birds.

Our experimental study supported the expected negative effect of increased egg white avidin concentration on chick morphology due to its extremely strong binding capacity to biotin (Green, 1975). Specifically, chicks hatching from lighter eggs supplemented with egg white avidin suffered from reduced structural body size (i.e. residual tarsus length) compared with those hatched from heavier, avidin-supplemented eggs. For chicks hatched from unmanipulated control eggs, this effect was lacking. These results are in agreement with those of Bonisoli-Alquati et al. (2010), who observed that an increased concentration of egg white avidin in natural eggs negatively affected tarsus length in yellow-legged gull (*Larus michahellis*) chicks. We believe that our observed egg weight-dependent negative effect of increased egg white avidin concentration on structural body size in freshly hatched quail chicks is linked with avidin's potential to affect *in ovo* biotin availability. Because biotin, which is released via biotinidase activity from egg yolk during embryogenesis, has multiple functions (e.g. it is strongly involved in the metabolism of proteins, lipids and carbohydrates (Couch et al., 1948; Dakshinamurti, 1994) and affects cell proliferation by influencing levels of gene expression (Manthey et al., 2002; Dakshinamurti, 2003, 2005), it is not surprising that biotin deficiency during embryogenesis in mammals resulted in inhibition of embryo development, embryo malformations and increased embryo mortality (Watanabe, 1993; Valenciano et al., 2002; Watanabe et al., 2009). There have only been a limited number of studies to date evaluating the role of *in ovo* biotin availability on embryogenesis in birds (Cravens et al., 1944; Whitehead et al., 1985). The most recent (Taniguchi and Watanabe, 2007) noted that concentration of free unbound biotin in chicken egg yolk increased immediately after fertilisation and, while most was incorporated by the embryo within the first 3-4 days, yolk concentration remained high during the first 11 days of embryo development. This free yolk biotin may penetrate through the vitelline membrane into the egg white in the later phases of embryogenesis, where it is probably trapped by avidin (Bush and White, 1989; White and Whitehead, 1987). Once the biotin is irreversibly bound to avidin, it becomes unavailable to the embryo until the day before hatching, when the embryo usually swallows the rest of the egg white and withdraws the yolk sac into its abdomen (White et al., 1992). There is strong evidence for the negative effect of biotin deficiency on growth performance in birds (Whitehead et al., 1985; Watkins, 1989; Zhu et al., 2012). Further, both *in vitro* (Zerega et al., 2001; Takechi et al., 2008) and *in vivo* studies (Hocking et al., 2013) have shown avidin suppressing biotin availability and development of chondroid and skeletal tissue. Hence, we assume that embryos developing in lighter avidin-treated eggs with less yolk (and thus less biotin) are more negatively affected by increased avidin as it traps diffused yolk biotin during the late phases of embryogenesis. As a result, tibia bone development is reduced compared with embryos developing in heavier avidin-manipulated eggs, where more yolk, and hence free biotin, is available. Although we were unable to measure biotin content in different-sized experimental eggs during this study, and multiple intrinsic factors may be involved during embryo development, our findings strongly support the assumption that egg white avidin favours embryo survival in nutritionally poorer small eggs, though at the expense of reduced structural body size.

In our experimental study, we evaluated the role of egg white avidin concentration on growth performance and innate immunity in LPS-treated and untreated quail chicks. We found that LPS treatment had no effect on total plasma complement activity, though it compromised chick growth performance, including both overall and structural body size. Our finding regarding the growth inhibiting effect of LPS treatment is in agreement with previous studies on poultry (Liu et al., 2015; Wang et al., 2015; Zheng et al., 2016). However, it is surprising that despite the evidence for avidin's involvement in regulation of tissue injury or viral transformation-induced inflammation processes (Korpela et al., 1983; Elo and Korpela, 1984), and the fact that antimicrobial and complement-related proteins are considered some of the main innate immunity effector molecules (Buchmann, 2014), we failed to observe any interactive effect of LPS treatment and egg white avidin treatment on total plasma complement activity. But, when we compared total plasma complement activity of control and LPS-treated 6-day-old chicks, we found that total plasma complement activity in large chicks was compromised due to increased egg white avidin concentration. In particular, plasma complement activity in 6-day-old chicks from eggs with experimentally increased egg white avidin concentrations decreased while body weight and residual tarsus length increased. The opposite effect was observed in chicks from control eggs. This suggests a trade-off in resource allocation strategies in embryos and/or hatchlings between growth and immunity.

Support for resource allocation in embryos from avidin-treated eggs is provided by Schmidt et al. (2015), who found that adult song sparrows (*Melospiza melodia*) reared under stressful condition had a reduced innate immune response, suggesting allocation strategies for immune system development under suboptimal rearing conditions. Similarly, Kriengwatana et al. (2013), in their study on zebra finches (*Taeniopygia guttata*), pointed out the importance of juvenile developmental condition on changes in immune and adult phenotype. As our findings suggest a negative effect of increased egg white avidin concentration on structural body size in quail hatchlings, we hypothesise that embryo developmental conditions in avidin-manipulated eggs were stressful and limited. This probably forced the embryos, and subsequently hatchlings, to allocate more resources into impaired development than immunity, resulting in a reduction in innate immunity function. Support for the negative effect of egg white avidin on innate immunity function can be found in Bonisoli-Alquati et al. (2010), who documented an impaired phytohaemaglutinin-induced immune response in 3-day-old yellow-legged gull chicks due to an increased concentration of egg white avidin.

In addition to the role played by egg white avidin as an essential egg white antimicrobial agent, protecting the embryo from invading microorganisms, the results of our experimental study provide a new insight into the role of egg white avidin as a strong precursor of growth and innate immune function and an essential developmental factor, affecting embryogenesis and having underlying phenotypic consequences in birds. However, according to our data, the effect of avidin was always dependent on the context of other factors such as egg weight or post-hatching immune challenge. We therefore conclude that future research should ask how these complex mechanisms are translated to fitness payoffs and what conditions favour higher maternal allocations of avidin into the egg under natural setup in precocial and altricial birds.

## MATERIALS AND METHODS

### Model species

We used the eggs of three separate lines of Japanese quail, a medium-sized galliform species, selected for the shape of their growth curve and varying in concentration of egg white avidin (for details see below) for experimental testing. Specifically, LG (low relative body weight gain between 11 and 28 days), HG (high relative body weight gain between 11 and 28 days) and C (an unselected control line) lines were used (see Hyánková et al., 2004 for details). All three lines were housed at the Institute of Animal Science in Prague-Uhrineves (Czech Republic).

### Pre-experimental measurement of egg characteristics

To assess line-specific differences in total egg weight, egg white volume and egg white avidin concentration, pre-experimental measurements were undertaken on freshly laid eggs (*n*=30 for each quail line); the third and fourth eggs in each laying sequence randomly were collected over two consecutive days. The eggs were cleaned with 70% ethanol and weighed to the nearest 0.01 g using a KERN CM50-C2M digital balance. Total egg weight (mean±s.e.) for HG, LG and C eggs was 12.6±0.76; 13.1±0.61 and 12.36±0.76 g, respectively, and showed no significant variation between lines (one-way ANOVA, *F*_(2,77)_=0.631; *P*=0.535). To obtain egg white volume (mean±s.d.), eggs were cracked, the egg whites and yolks carefully separated and each egg white weighed to the nearest 0.001 g. These weights were converted to volume (nearest 0.01 ml) based on the measurement of 1000 µl of egg white for which weight was 0.965 g. This method enabled us to avoid possible inaccuracy in direct volume measurement caused by egg white viscosity. Line-specific egg white volume was calculated based on egg white weight multiplied by a coefficient of 0.965, corresponding to volumetric mass density of egg white for each quail line. Egg white volume (mean±s.e.) for HG, LG and C line eggs was 6.7±0.48 ml, 6.9±0.46 ml and 6.8±0.55 ml, respectively, and showed no significant variation between lines (one-way ANOVA; *F*_(2,77)_=0.361; *P*=0.702). Each fresh egg white was subsequently analysed for avidin concentration (mean±s.e., in order to calculate line-specific total egg white avidin concentration). Egg white avidin concentration differed significantly between quail lines (one-way ANOVA, *F*_(2,59)_=21.803; *P*<0.0001), with line-specific egg white avidin concentrations (mean±s.e.) (µg/ml) for HG, LG and C line eggs of 0.88±0.64; 2.75±1.24 and 1.96±1.05, respectively.

### Analysis of egg white avidin concentration

Egg white avidin concentration (µg/ml) was based on a slightly modified version of the 96-well plate method of Shawkey et al. (2008). We diluted each egg white sample 10-fold in carbonate-bicarbonate buffer (made from Sigma-Aldrich C3041 capsules, following the manufacturer’s instructions). We then added 100 µl of carbonate-bicarbonate buffer to each well (except the first, fifth and ninth well in each row) along rows one to 11 of a Nunc MaxiSorp^®^ flat-bottom 96-well plate. In order to ensure accurate pipetting of undiluted and diluted egg white samples, we used GENO-DNA S pipette tips (CS960 9405120, Thermo Fisher Scientific), specially designed for viscous liquids. Sample absorbances were measured at 450 nm using a TECAN Infinite^®^ 200 PRO UV/Vis microplate reader (Tecan Group, Männedorf, Switzerland). Avidin concentrations (considering egg white serial dilutions) were determined by interpolating from a standard curve for each plate using GraphPad Prism 5 Software (inter-assay and intra-assay coefficients of variability were 14.8% and 3.7%, respectively).

### Manipulation experiment

The manipulation experiment consisted of three replicates of 3×150 experimental eggs. For each experimental replicate, we used freshly laid eggs (*n*=150; 50 HG, 50 LG, 50 C quail line eggs) from 40 line-specific breeding hens (i.e. each hen producing a maximum of two eggs for our purposes), using the third and fourth eggs in each laying sequence collected randomly over two consecutive days. These were placed into portable plastic boxes and stored in an air-conditioned dark room at 21°C for no more than 24 h. The 50 eggs of each quail line were then randomly divided into two groups: (i) those manipulated *in ovo* with avidin=AVIDIN+ eggs, and (ii) a control group with sterile phosphate buffer saline (PBS)-injected *in ovo*=PBS+ eggs. After cleaning with 70% EtOH, AVIDIN+ eggshells were gently perforated with a 0.33×12.7 mm Micro-Fine Plus needle (5 mm below the equatorial region, toward the blunt end) and, using a 1 ml insulin syringe, injected with 50 µl of a solution containing purified lyophilised egg white avidin (A9275, Sigma-Aldrich) diluted in sterile PBS (GIBCO^®^, pH 7.2, Invitrogen). The quantity of injected avidin corresponded to one standard deviation of line-specific mean egg white avidin content (for HG line eggs, 4.3 µg; for LG line eggs, 8.6 µg; for C line eggs, 7.2 µg of PBS-diluted avidin). PBS+ eggs were processed identically, but injected with 50 µl of sterile PBS only. Needle perforations in the eggshells were sealed using a gel-based adhesive (Loctite-Super Attack, Henkel, Stamford, USA), and the eggs were placed into an OvaEasy 190 Advance incubator with automatic egg turning (Brinsea Products, Titusville, USA).

Optimal incubation conditions for Japanese quail eggs are 37.6°C and 58% relative humidity, with relative humidity increased to 80% during the egg hatching period (see Pedroso et al., 2006). To control for chick identity, each egg was placed into a net sack 2 days before hatching (Hořák and Albrecht, 2007). An egg was assigned as fertile post-treatment following successful hatching or in the presence of a cicatricula or dead embryo inside an unhatched egg (Sellier et al., 2005). For unhatched eggs with dead embryos, stage of embryo mortality during the first (early) and third (late) trimester of development was assigned based on the developmental stages described by Hamburger and Hamilton (1992). Specifically, death of an embryo in the first 6 days of incubation (stages 1 to 29) was assigned as early embryonic mortality and from 12 to 17 days of incubation (stages 38 to 46) as late embryonic mortality (see Hamburger and Hamilton, 1992 for details).

### Post-hatching experimental procedures

Chick body weight (±0.1 g) and length of both tarsi (±0.1 mm) was measured immediately after hatching using a Kern CM50-C2M digital balance and digital callipers. The same morphometric parameters were measured at 3-day intervals throughout the 15-day post-hatching period (all chicks were measured six times over 15 days). To control for chick identity, each was individually marked with a coloured plastic ring with a unique number.

To induce the chick's innate immunity, 3-day-old chicks from each line were randomly divided into two groups. Each chick in one group was injected intramuscularly with 80 µl LPS solution (dose equivalent to 8 mg LPS per kg of body weight) dissolved in sterile PBS using a 1 ml insulin syringe (hereafter referred to as LPS-treated chicks). As body weight differed considerably among 3-day-old chicks from different quail lines, doses were adjusted to 0.104, 0.208 and 0.184 mg LPS for HG, LG and C line chicks, respectively. Chicks in the second group (i.e. control PBS-treated chicks) were injected with 80 µl of sterile PBS only.

Blood samples from a brachial vein puncture were collected into 40 µl heparinised capillaries (Keraglass, Otvovice, Czech Republic) 6 days after hatching (i.e. 3 days after LPS/PBS treatment) in order to measure total plasma complement activity. The capillaries were placed on ice and centrifuged for 10 min at relative centrifugal force (RCF)=2000×***g*** within 1 h using a refrigerated capillary centrifuge (Eppendorf). Plasma supernatants were separated and transferred into 1.5 ml sterile cryotubes (Merck KGaA, Darmstadt, Germany, Czech Republic) and stored at −80°C until analysis.

### Analysis of plasma complement activity

Plasma complement activity was measured using a modified version of the method outlined by Buchtíková et al. (2011). Briefly, total plasma complement activity was determined through a bioluminescence-based method that used transformed *Escherichia coli* K12 with the luxABCDE gene on the bacterial plasmid, which expresses bacterial luciferase (Lux) and its substrate, a long-chain aldehyde. The time in seconds required for 50% viability of *E. coli* was evaluated using kinetic curves corresponding to the plasma complement activity of each plasma sample. There is an inverse relationship between time of *E. coli* viability and total plasma complement activity, with a shorter time representing higher total plasma complement activity.

### Statistical analysis

GLMMs were used to analyse the data, with the identity of the experimental replicate included as a random effect in all models to account for this source of variation. Further, we applied strain-specific z-score transformation to all continuous variables included in individual models (detailed below) in order to maintain constant variation and zero mean for all three quail lines used in the experiment. This allowed us to avoid poor fit of the models due to inconstant variation of residuals between individual quail lines. Importantly, line-specific scaling of continuous variables resulted in less complex minimal adequate models, because the significance of morphometric parameters that are known to differ between quail lines (Hyánková et al., 2004, 2015), but have effects irrelevant to our experiment, was offset by this step. Because data on tarsus length and body weight were highly correlated (Pearson's r=0.320, *P*<0.0001), body weight was assumed to reflect overall body size and residuals from linear regression between body weight and tarsus length were assumed to reflect structural body size. These were then used as response variables in all statistical models to achieve orthogonality and to avoid multicollinearity in fitting models.

The effect of avidin treatment on hatching success was analysed using binary GLMM (binomial distribution, logit link). First, hatched versus unhatched eggs were coded as binary response variables. The same approach was used during separate analyses of early and late embryonic mortality (see Materials and Methods for more detail). The binomial GLMM approach described in Aebischer (1999) was then used to analyse post-hatching survival rate. Briefly, the number of unsuccessful days (0 for hatchlings that survived the 15-day post-hatching experimental period; 1 for those that died during the post-hatching experimental period) versus the number of successful days was computed for all individuals and included as a binomial response variable in the GLMM.

Morphological traits and post-hatching growth performance were analysed by assessing whether overall body size (i.e. body weight) and structural body size (i.e. body weight versus tarsus length linear regression residuals) after hatching differed between treatment groups by including these variables as a response in a GLMM. Avidin treatment, line identity, egg weight and all two-way interactions were included as explanatory variables. Scaled body weight and residual tarsus length following z-score transformation were calculated separately for each of the six age classes and three quail lines. The effect of chick age was included as a third order polynomial as both a fixed and random effect in the model. The random structure of these models accounts for the same individual being measured repeatedly.

The effect of LPS treatment on growth performance was analysed using a GLMM with difference in body weight and residual tarsus length between 3-day-old and 6-day-old chicks as a response variable. LPS treatment, avidin treatment, line identity and their two-way interactions were included as explanatory variables in the GLMM.

Finally, we assessed the extent to which total plasma complement activity (included as a response variable) was affected by avidin treatment, line identity, body weight and residual tarsus length of 6-day-old chicks, with two-way interactions between these variables included as explanatory variables in the GLMM.

For all the above GLMMs, we assessed the degree to which line specific scaling affected model outcome. In all cases, we found that the model outcomes obtained using unscaled data were consistent with, or less conservative than, outcomes of models fitted for scaled data.

All analyses were performed using R software v. 3.0.2 (R Development Core team, 2013). The models were fitted using the lme4 package running under R (Bates et al., 2013). Predictions±95% bootstrap confidence intervals (10,000 steps) of relevant terms were generated using the ez package in R (Lawrence, 2012).
